# Intracerebral transplantation of interleukin 13-producing mesenchymal stem cells limits microgliosis, oligodendrocyte loss and demyelination in the cuprizone mouse model

**DOI:** 10.1186/s12974-016-0756-7

**Published:** 2016-11-09

**Authors:** Debbie Le Blon, Caroline Guglielmetti, Chloé Hoornaert, Alessandra Quarta, Jasmijn Daans, Dearbhaile Dooley, Evi Lemmens, Jelle Praet, Nathalie De Vocht, Kristien Reekmans, Eva Santermans, Niel Hens, Herman Goossens, Marleen Verhoye, Annemie Van der Linden, Zwi Berneman, Sven Hendrix, Peter Ponsaerts

**Affiliations:** 1 Laboratory of Experimental Hematology, University of Antwerp, Universiteitsplein 1, 2610 Antwerp, Belgium; 2 Vaccine and Infectious Disease Institute, University of Antwerp, Universiteitsplein 1, 2610 Antwerp, Belgium; 3 Bio-Imaging Laboratory, University of Antwerp, Universiteitsplein 1, 2610 Antwerp, Belgium; 4 Department of Morphology, Biomedical Research Institute, Hasselt University, Agoralaan building C, 3590 Diepenbeek, Belgium; 5 Center for Statistics, I-Biostat, Hasselt University, Agoralaan building D, 3590 Diepenbeek, Belgium; 6 Centre for Health Economic Research and Modeling Infectious Diseases (Chermid), University of Antwerp, Universiteitsplein 1, 2610 Antwerp, Belgium; 7 Experimental Cell Transplantation Group, Laboratory of Experimental Hematology, Vaccine and Infectious Disease Institute (Vaxinfectio), University of Antwerp, Campus Drie Eiken (CDE-S6.51), Universiteitsplein 1, 2610 Antwerp, Wilrijk Belgium

**Keywords:** Interleukin 13, Mesenchymal stem cells, Neuroinflammation, Transplantation, Magnetic resonance imaging

## Abstract

**Background:**

Promoting the neuroprotective and repair-inducing effector functions of microglia and macrophages, by means of M2 polarisation or alternative activation, is expected to become a new therapeutic approach for central nervous system (CNS) disorders in which detrimental pro-inflammatory microglia and/or macrophages display a major contribution to the neuropathology. In this study, we present a novel in vivo approach using intracerebral grafting of mesenchymal stem cells (MSC) genetically engineered to secrete interleukin 13 (IL13-MSC).

**Methods:**

In the first experimental setup, control MSC and IL13-MSC were grafted in the CNS of eGFP^+^ bone marrow chimaeric C57BL/6 mice to histologically evaluate IL13-mediated expression of several markers associated with alternative activation, including arginase1 and Ym1, on MSC graft-recognising microglia and MSC graft-infiltrating macrophages. In the second experimental setup, IL13-MSC were grafted on the right side (or on both the right and left sides) of the *splenium* of the *corpus callosum* in wild-type C57BL/6 mice and in C57BL/6 CX_3_CR1^eGFP/+^CCR2^RFP/+^ transgenic mice. Next, CNS inflammation and demyelination was induced by means of a cuprizone-supplemented diet. The influence of IL13-MSC grafting on neuropathological alterations was monitored by non-invasive *T*_2_-weighted magnetic resonance imaging (MRI) and quantitative histological analyses, as compared to cuprizone-treated mice with control MSC grafts and/or cuprizone-treated mice without MSC injection.

**Results:**

In the first part of this study, we demonstrate that MSC graft-associated microglia and MSC graft-infiltrating macrophages are forced into alternative activation upon grafting of IL13-MSC, but not upon grafting of control MSC. In the second part of this study, we demonstrate that grafting of IL13-MSC, in addition to the recruitment of M2 polarised macrophages, limits cuprizone-induced microgliosis, oligodendrocyte death and demyelination. Furthermore, we here demonstrate that injection of IL13-MSC at both sides of the splenium leads to a superior protective effect as compared to a single injection at one side of the splenium.

**Conclusions:**

Controlled and localised production of IL13 by means of intracerebral MSC grafting has the potential to modulate cell graft- and pathology-associated microglial/macrophage responses, and to interfere with oligodendrocyte death and demyelinating events in the cuprizone mouse model.

## Background

Detrimental inflammatory responses in the central nervous system (CNS) are a hallmark of various neurodegenerative pathologies, with multiple sclerosis (MS), stroke and traumatic brain or spinal cord injury being excellent examples of the complex interplay between CNS-resident microglia and lesion-infiltrating leukocytes [1–4]. Although microglia and CNS-invading peripheral monocytes both act as phagocytic cells of the brain, it is currently becoming accepted that these two cell types are more distinct than generally assumed [2, 5]. This is supported by observations in various models of neuroinflammation in which microglia and bone marrow (BM)-derived macrophages may be present at different time points, may have a different spatial distribution and, more importantly, may display different functions and/or phenotypes during the course of neuroinflammation and neurodegeneration [5]. Nevertheless, in the early stage of neuroinflammatory responses, lesion-associated microglia and/or macrophages are generally described as ‘classically or M1 activated’, i.e. displaying a phenotype and function comparable to in vitro LPS- and/or IFNγ-activated macrophages [6]. M1 activated macrophages are thus highly pro-inflammatory and cytotoxic, with their main effector functions being the destruction and removal of damaged or infected cells. From an emerging therapeutic point of view, it is suggested that forced ‘alternative or M2 activation’ of lesion-associated microglia and/or macrophages during the development of inflammatory responses can have a beneficial effect on disease outcome [5]. M2-activated macrophages are thus suggested to be anti-inflammatory, with their main effector functions being protection and regeneration of injured or diseased tissue. Although dependent on the stimuli applied, several directions of alternative macrophage activation can be achieved in vitro and in vivo [6]. In this study, we aimed to investigate the potential of interleukin 13 (IL13) [7] to modulate microglia and/or macrophage activation in vivo in an attempt to influence pathology-associated neuroinflammation.

One of the major challenges in current CNS immunomodulation research is how to effectively deliver therapeutic proteins (*in casu* IL13) directly to the site of neuroinflammation. While several methods of delivery can be implemented [8], including (i) direct protein injection, (ii) non-viral and viral gene therapy and (iii) implantation of genetically engineered cellular grafts, each of them has specific advantages and disadvantages. Whereas direct protein injection would be the most straightforward, it would require multiple injections as, in the case of IL13, sustained therapeutic protein expression may be essential. Alternatively, mechanical or chemical methods (e.g. electroporation, ultrasound or lipoplexes) may be applied to transfer plasmid DNA encoding the therapeutic protein of interest into inflammatory cells at the site of neuroinflammation. Nevertheless, these techniques are still poorly efficient and need further optimisation for in vivo application. On the other hand, gene transfer in the CNS by means of viral vectors is highly efficient in rodents but remains controversial in terms of clinical translation to humans, despite many efforts undertaken to control gene insertion, protein expression and/or unwanted immune reactions. Lastly, transplantation of genetically engineered (stem) cell populations is an emerging methodological approach for in situ delivery of therapeutic proteins [9, 10]. Preceding work by our group has already extensively compared the in vivo behaviour of neural stem cell (NSC) and mesenchymal stem/stromal cell (MSC) grafts upon implantation in the CNS of mice [11–16]. Based on our published reports, we have a strong preference for MSC as a cellular carrier to deliver therapeutic proteins due to their relatively easy ex vivo culture, susceptibility for genetic modification and their more robust survival upon grafting in CNS tissue compared to NSC. In this study, we aim to investigate whether in situ grafting of MSC genetically engineered to express IL13 can influence neuroinflammatory responses, both on the level of cell graft-associated inflammatory responses and on the level of pathology-associated inflammatory responses.

In order to address these questions, we investigated the behaviour of control MSC and IL13-expressing MSC grafts both under healthy and under inflammatory CNS conditions. For the latter, we used the well-established cuprizone (CPZ) mouse model of CNS inflammation, oligodendrocyte death and subsequent demyelination [17]. Furthermore, in order to separately investigate the behaviour of brain-resident microglia and CNS-invading peripheral macrophages, part of the experiments presented in this study were performed in the CX_3_CR1^eGFP/+^ CCR2^RFP/+^ transgenic mouse model or in eGFP^+^ bone marrow (BM) chimaeric mice.

## Methods

### Mice

Wild-type C57BL/6 mice were obtained via Charles River Laboratories (strain code 027). Transgenic C57BL/6-eGFP mice (strain code 003291), CX_3_CR1^eGFP/eGFP^ mice (strain code 005582) and CCR2^RFP/RFP^ mice (strain code 017586) were obtained via Jackson Laboratories. CX_3_CR1^eGFP/+^ CCR2^RFP/+^ mice were obtained by breeding CX_3_CR1^eGFP/eGFP^ mice with CCR2^RFP/RFP^ mice. During the entire study, the mice were kept in the animalarium of the University of Antwerp (UA) under normal day-night cycle (12/12) with free access to food and water. All animal experimental procedures were approved by the Ethics Committee for Animal Experiments of the UA (approval no. 2011–13 and 2012–39).

### Lentiviral vector production

The pCHMWS-IL13-IRES-Pac lentiviral vector (LVv) plasmid was constructed by replacing the eGFP cDNA insert (SpeI/XbaI digest) from the pCHMWS-eGFP-IRES-Pac plasmid (provided by the Leuven viral vector core, Molmed, KULeuven, Belgium) with the IL13 cDNA (NcoI/NheI digest) from the pORF-mIL13 plasmid (InvivoGen) using standard subcloning techniques. Before proceeding to LVv production, the pCHMWS-IL13-IRES-Pac plasmid was electroporated in K562 cells followed by stable selection by addition of puromycin to the culture medium. Expression of IL13 was confirmed by a murine IL13 ELISA (Peprotech) using the supernatant of stably transfected puromycin-resistant K562 cells. Following confirmation of IL13 expression and Pac functionality, LVv production was outsourced to the Leuven viral vector core [18, 19].

### Culture and genetic engineering of MSC

In this study, we used a previously established and characterised C57BL/6 mouse BM-derived MSC line (further named as parental MSC) [20] and a derivative thereof, genetically engineered to express the blue fluorescent protein (further named as blue fluorescent protein (BFP)-MSC) [13]. For expansion, both MSC lines were cultured in standard cell culture plasticware (well plates and/or culture flasks) in ‘complete expansion medium’ (CEM) [21] consisting of Iscove’s modified Dulbecco’s medium (IMDM; Lonza) supplemented with 8% fetal bovine serum (FBS; Invitrogen), 8% horse serum (HS; Invitrogen), 200U/ml + 100 μg/ml penicillin/streptomycin (Invitrogen) and 1 μg/ml amphotericin B (Invitrogen). Culture medium for BFP-MSC was further supplemented with 1 μg/ml puromycin (InvivoGen). MSC cultures were split 1:5 twice a week using 0.05% trypsin-EDTA (Invitrogen) for cell detachment. Both the parental MSC line and the BFP-MSC line were transduced with the pCHMWS-IL13-IRES-Pac LVv, according to previously optimised procedures [13, 21]. Following puromycin selection, the resulting engineered MSC lines were further named as IL13-MSC and IL13/BFP-MSC. Culture medium for both IL13-MSC and IL13/BFP-MSC was further supplemented with 5 μg/ml puromycin. Expression of IL13 was confirmed by a murine IL13 ELISA, demonstrating a secretion of 184 ± 44 ng IL13/6 × 10^4^ cells/24 h by IL13-MSC and 76 ± 3 ng IL13/6 × 10^4^ cells/24 h by IL13/BFP-MSC. No IL13 secretion was detected in BFP-MSC cultures. IL13-MSC were used in three experiments, and IL13/BFP-MSC were used in one experiment out of four in the cuprizone mouse model. To avoid confusion, both IL13-producing cell lines will be referred to as IL13-MSC further in text.

### Bone marrow transplantation experiments

In order to discriminate between microglia and macrophages, eGFP^+^ BM chimaeric mice were generated as previously described [13]. Briefly, wild-type C57BL/6 mice received 10Gy total body irradiation using an XRAD320 small animal irradiation device (Precision X-Ray). For this, groups of five non-anesthetised mice were placed in a single cage within the whole irradiation field (without head protection). Six hours post-irradiation, a single intravenous injection of total eGFP^+^ BM cells (1.5 × 10^6^ cells in 100 μl phosphate buffered saline) was administered via the tail vein. Total BM cells were isolated from 8-week-old C57BL/6-eGFP mice by flushing dissected femurs and tibias with sterile phosphate buffered saline (PBS). Before administration, total BM cells were filtered over a 70-μm sterile mesh (Becton Dickinson), centrifuged and suspended in PBS. During recovery, mice were treated with enrofloxacin (1 μl/ml; Baytril 10%; Bayer) added to the drinking water for 8 weeks post-irradiation.

### Cell implantation experiments

All surgical experiments were performed under sterile conditions, as previously described [22–24]. Briefly, mice were anesthetised by an intraperitoneal injection of a ketamine (80 mg/kg, Pfizer) + xylazine (16 mg/kg, Bayer Health Care) mixture in PBS and placed in a stereotactic frame (Stoelting). A midline scalp incision was made and a hole was drilled in the skull using a dental burr drill (Stoelting) at the following coordinates relative to *bregma:* (i) 2.3 mm *dextra* for injections in healthy mice, or (ii) 0.3 mm *dextra* at 1.6 mm *posterior* for injections in CPZ-treated mice or (iii) 0.3 mm *dextra* and 0.3 mm *sinistra* at 1.6 mm *posterior* for double injections in CPZ-treated mice. Next, an automatic microinjector pump (kdScientific) with a 10 μl Hamilton syringe was positioned above the exposed *dura*. A 30-gauge needle (Hamilton), attached to the syringe, was placed through the intact *dura* to a depth of 2.3 mm for injections in healthy mice (directly underneath the *capsula externa*) and to a depth of 1.3 mm in CPZ-treated mice (directly within the right side or both left and right sides of the *splenium* of the *corpus callosum)*. After 2 min of pressure equilibration, a suspension of 2 × 10^4^ MSCs in a volume of 0.4 μl was injected in CPZ-treated mice or a suspension of 2 × 10^5^ MSCs in a volume of 2 μl was injected in healthy mice. The needle was retracted after another 4 min to allow pressure equilibration and to prevent backflow of the injected cell suspension. Next, the skin was sutured (Vicryl, Ethicon) and 100 μl of a 0.9% NaCl solution (Baxter) was administered subcutaneously in order to prevent dehydration while mice were placed under a heating lamp to recover.

### Cuprizone mouse model

For induction of CNS inflammation and demyelination, mice received standard rodent chow mixed with 0.2 % *w*/*w* CPZ (Sigma-Aldrich) for 4 weeks between the ages of 8 and 12 weeks, as previously described [14, 25–27].

### Magnetic resonance imaging acquisition

For the first study setup, in which a single injection on the right side of the splenium was performed in CPZ-treated mice, in vivo imaging experiments were conducted at 400 MHz on a 9.4T Bruker Biospec system (Biospec 94/20 USR, Bruker Biospin) using a standard Bruker cross coil setup, with a quadrature volume coil for excitation and a quadrature mouse surface coil for signal detection. For the second study setup, in which a double injection on both the right side and the left side of the splenium was performed in CPZ-treated mice, in vivo imaging experiments were conducted at 400 MHz on a 7T Pharmascan MR scanner (Bruker, Germany). This system is equipped with a standard Bruker cross coil setup, with a quadrature volume coil for excitation and an array mouse surface coil for signal detection. During imaging, mice were anesthetised using 2% isoflurane (Isoflo®, Abbot Laboratories Ltd.) in a mixture of 30% O_2_ and 70% N_2_O at a flow rate of 600 ml/min. Mice were fixed in an animal restrainer with ear bars and a tooth bar. Respiratory rate was continuously monitored, and body temperature was measured and maintained constant at 37 °C using a feedback coupled warm air system (MR compatible Small Animal Monitoring and Gating System, SA instruments, Inc.). *T*_2_ values were acquired with the multi-slice multi-echo (MSME) sequence that is based on the Carr-Purcell-Meiboom-Gill (CPMG) sequence, where transverse magnetization of a 90° pulse is refocused by a train of 180° pulses generating a series of echoes. For the study setup with a single injection on the right side of the splenium, the following imaging parameters were used: number of averages (NA) = 1; number of slices (NS) = 6 with a slice thickness of 0.4 mm and an interslice thickness of 0.4 mm; number of echoes = 10 with echo spacing = 8.5 ms (echo times (TE) being 8.5; 17; 25.5; 34; 42.5; 51; 59.5; 68; 76.5; 85); a repetition time (TR) = 4000 ms; field of view (FOV) = 2.0 × 2.0 cm; matrix size = 256 × 256 (this yields an effective in-plane resolution of 0.078 × 0.078 mm). The total acquisition time per analysis was 12 min 48 s. For the study setup with double injection on both the right and left sides of the splenium, the following parameters were used: NA = 1; NS = 6 with a slice thickness of 0.4 mm and an interslice thickness of 0.4 mm; number of echoes = 10 with echo spacing = 8.7 ms (TE being 8.7; 17;4; 26.1; 34.8; 43.5; 52; 60.9; 69.6; 78.3; 87); TR = 4000 ms; FOV = 2.5 × 2.5 cm; matrix size = 256 × 256 (this yields an effective in-plane resolution of 0.098 × 0.098 mm). The total acquisition time per analysis was 12 min 48 s.

### Magnetic resonance imaging processing

*T*_2_ maps were generated with custom-built programmes written in MATLAB (MATLAB R2011b, The MathWorks Inc.) using a monoexponential fit function [*y* = *A* + C*exp (−t/*T*_2_)], where *A* = absolute bias, *C* = signal intensity and *T*_2_ = transverse relaxation time. Regions of interest (ROIs) were drawn manually on the *T*_2_-weighted images, according to a mouse brain atlas, with AMIRA software (Mercury Computer systems) and regional average *T*_2_ values were calculated. ROIs included the external capsule (EC) and the *splenium* of the *corpus callosum* (CC), which was further divided in two parts in respect to the brain’s midline and later referred as the right and the left sides of the *splenium*.

### Immunofluorescence analysis

All immunofluorescence analyses were performed according to previously described procedures [22, 23]. Mice were transcardially perfused with 0.9 % NaCl solution followed by 4% paraformaldehyde (PFA) solution. Next, brains were isolated and further fixed in 4 % PFA for 3 h, then dehydrated through a sucrose gradient of 5, 10 and 20 %. Afterwards, brain tissue was snap-frozen in liquid nitrogen and kept at −80 °C until further processing. Ten-micrometer-thick cryosections were made using a microm HM500 cryostat. Immunofluorescence staining was performed on brain slides using the following antibody combinations: a primary goat anti-MBP antibody (1 μg/ml, Santa Cruz, sc-13914) with a secondary donkey anti-goat Alexa Fluor 350 antibody (20 μg/ml, Invitrogen,A21081); a primary rabbit anti-Iba1 antibody (1 μg/ml, Wako, 019-19741) with a secondary donkey anti-rabbit Alexa Fluor 555 (2 μg/ml, Invitrogen, A31572); a primary rabbit anti-GFAP antibody (4.5 μg/ml, Abcam, ab7779) with a secondary donkey anti-rabbit Alexa Fluor 555 antibody; a primary rat anti-F4/80 antibody (4 μg/ml, AbD serotec, MCA497GA) with a secondary goat anti-rat Cy5 antibody (10 μg/ml, Invitrogen, A10525); a primary rat anti-MHCII antibody (2.5 μg/ml, eBioscience, 14-5321-82) with a secondary goat anti-rat Alexa Fluor 555 or goat anti-rat Alexa Fluor 350 antibody (10 μg/ml, Invitrogen, A21093); a primary goat anti-Arg1 antibody (2 μg/ml, Santa Cruz, sc-18354) with a secondary donkey anti-goat Alexa Fluor 555 (10 μg/ml, Invitrogen, A21432) or donkey anti-goat Cy5 antibody (10 μg/ml, Abcam, AB6566); a primary rabbit anti-Ym1 antibody (0.4 μg/ml, Stemcell Technologies, 01404) with a secondary donkey anti-rabbit Alexa Fluor 555 antibody; a primary chicken anti-MBP antibody (0.5 μg/ml, Millipore, AB9348) with a secondary donkey anti-chicken Cy3 antibody (7.5 μg/ml, Jackson Immunoresearch, JIR703-166-155); and a primary mouse anti-CC1 antibody (0.5 μg/ml, Millipore, OP80) with a secondary goat anti-mouse Alexa Fluor 555 antibody (10 μg/ml, Invitrogen, A21425).

### Histological quantification

Quantitative phenotypic analyses of microglia and macrophage and responses following MSC grafting in bone marrow chimaeric mice were performed using TissueQuest immunofluorescence analysis software (TissueGnostics GmbH, v3.0), as previously described by our group [12, 13, 15, 23]. Briefly, for each of the slides analysed, the MSC graft site was manually delineated based on nuclear TOPRO-3 staining. The MSC graft site border was then determined as a region extending 85 μm from the MSC graft site. According to previously established procedures, the following parameters were determined: the cellular density of (i) eGFP^+^ macrophages (three slides per cell graft analysed), (ii) MHCII^+^ eGFP^+^ macrophages (one slide per cell graft analysed), (iii) Arg1^+^ eGFP^+^ macrophages (one slide per cell graft analysed), (iv) Iba1^+^ cells (one slide per graft analysed) and (v) MHCII^+^ Arg1^+^ cells (one slide per cell graft analysed). Quantitative analyses of microglial inflammation and/or demyelination in the cuprizone mouse model were performed using NIH ImageJ analysis software (v1.47), according to previously established procedures [13, 15, 23]. Briefly, the following parameters were determined for the whole *splenium* and the right and the left sides of the *splenium*: (i) percentage of eGFP coverage (three slides per mouse brain analysed) and (ii) percentage of MBP coverage (one to three slides per mouse brain analysed). In addition, the cellular density of CC1^+^ oligodendrocytes was determined using TissueQuest software (one slide per mouse brain analysed).

### Statistical analyses

For the results obtained following MSC implantation in the CNS of healthy eGFP^+^ bone marrow chimaeric mice, the following statistical analyses were applied. (i) Differences in cellular density of MHCII- and Arg1-expressing eGFP^−^ microglia and eGFP^+^ macrophages within the implant site and the implant border of BFP-MSC and IL13-MSC grafts were analysed using Wilcoxon tests. (ii) Differences in cellular density of all subgroups present in the implant site and the implant border of BFP-MSC and IL13-MSC grafts, based on eGFP, MHCII and Arg1 expression were analysed using Wilcoxon tests. However, because groups were very small (*n* = 5 or 6), the *p* value produced by this test is bounded by a non-significant minimal value. We therefore used *t* tests when normality was not rejected. We here rely on the normality assumption, since normality tests suffer from low power in this low sample size setting. *p* values were corrected for multiple testing using the false discovery rate method [28]. For the results obtained following MSC grafting in the cuprizone mouse model, the following statistical analyses were applied. (i) Comparisons of mean *T*_2_-relaxation times of the left, right and whole *splenium* of the CC, and the EC between healthy mice, CPZ-treated mice, BFP-MSC grafted CPZ-treated mice and IL13-MSC grafted CPZ-treated mice (right side injection) were analysed using a two-way ANOVA, with the obtained *p* values being corrected for multiple testing using the Tukey HSD post hoc test. In this way, possible differences between the three experiments are taken into account. (ii) Comparisons of mean *T*_2_ relaxation times of the left, right and whole splenium, and the EC between healthy mice, CPZ-treated mice and IL13-MSC injected mice (right and left side injection) were analysed using non-parametric Wilcoxon tests, with the given *p* values being corrected for multiple testing using the false discovery rate method. (iii) Comparisons of the percentages of eGFP and MBP coverage in the left, right and whole *splenium* of CX_3_CR1^eGFP/+^ CCR2^RFP/+^ mice between healthy mice, CPZ-treated mice, BFP-MSC grafted CPZ-treated mice and IL13-MSC grafted CPZ-treated mice (right side injection) were analysed by using GEE, with the given *p* values being corrected for multiple testing using the false discovery rate method [29]. (iv) Comparisons of MBP coverage percentages and CC1+ cell density in the left, right and whole splenium between healthy mice, CPZ-treated mice and IL13-MSC injected mice (right and left side injection) were analysed using a Wilcoxon rank sum test, with the given *p* values being corrected for multiple testing using the false discovery rate method. (v) The dependence between eGFP and MBP coverage percentage (right side injection), eGFP coverage percentage and *T*_2_ relaxation times (right side injection), MBP coverage percentage and *T*_2_ relaxation times (right side injection), MBP coverage percentage and CC1+ cell density (right and left side injection), MBP coverage percentage and *T*_2_ relaxation times (right and left side injection) and CC1+ cell density and *T*_2_ relaxation times (right and left side injection) was measured using the Spearman correlation coefficient. A bootstrap procedure was used to construct 95 % confidence intervals. Correlation coefficients and confidence intervals are stated in the results section.

## Results

### Transplantation of IL13 producing MSC in the CNS locally induces expression of markers associated with alternative activation in microglia and macrophages

**Fig. 1:**
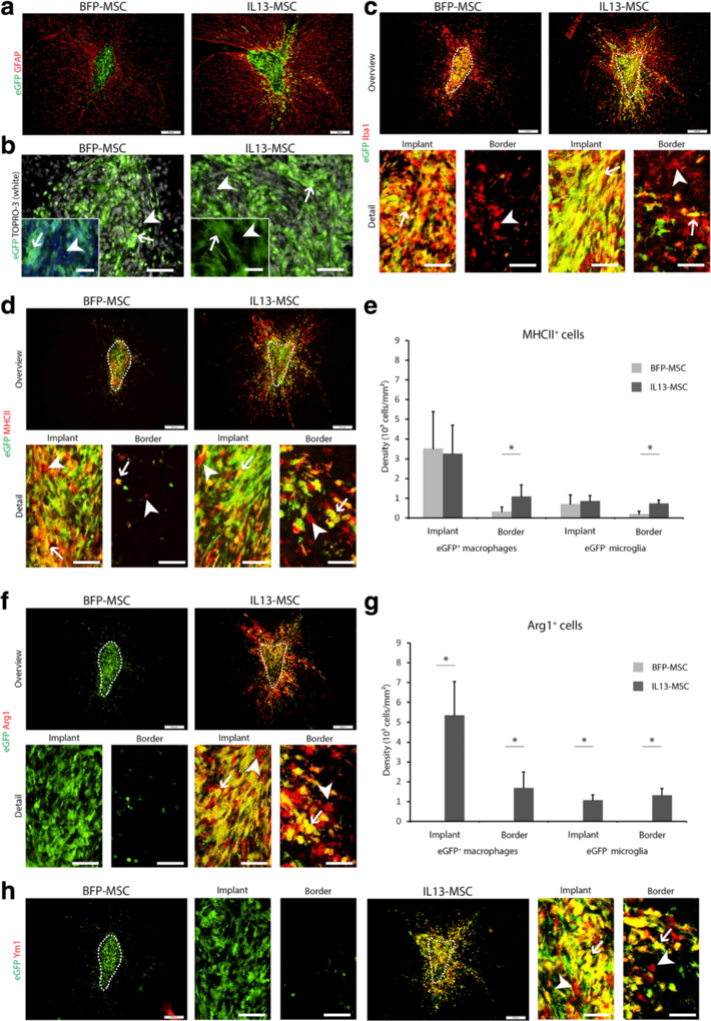
Analysis of BFP-MSC and IL13-MSC graft remodelling in the CNS of eGFP^+^ bone marrow chimaeras. **a** Overview of a BFP-MSC and IL13-MSC graft. *Green*: eGFP fluorescence from bone marrow-derived macrophages indicating infiltration at the graft. *Red*: GFAP staining indicates astrogliosis around the graft. *Scale bars* indicate 200 μm. **b** Detail of the BFP-MSC and IL13-MSC graft. *White*: TOPRO-3 marking all nuclei. *Green*: eGFP fluorescence from graft-infiltrating macrophages (*arrows*). eGFP^−^ nuclei (*arrowheads*) are grafted MSC or (few) graft-infiltrating microglia. *Scale bars* indicate 50 μm. *Insets* show BFP fluorescence (*blue*) or no fluorescence (*black*) from grafted BFP-MSC or IL13-MSC (*arrowheads*). *Green*: eGFP fluorescence from graft-infiltrating macrophages (*arrows*). *Scale bars* indicate 20 μm. **c** Overview of a BFP-MSC and IL13-MSC graft. *Green*: eGFP fluorescence from graft-infiltrating macrophages. *Red*: Iba1 staining marks both eGFP^−^ microglia and eGFP^+^ macrophages. *Scale bars* indicate 200 μm. Details of the BFP-MSC and IL13-MSC implant and border show Iba1^+^eGFP^+^ peripheral macrophages mainly within the implant (*arrows*) and Iba1^+^eGFP^−^ microglia mainly within the border (*arrowheads*). *Scale bars* indicate 50 μm. **d** Overview of a BFP-MSC and IL13-MSC graft. *Green*: eGFP fluorescence from graft-infiltrating macrophages. *Red*: MHCII-expressing eGFP^−^ brain-resident microglia and eGFP^+^ peripheral macrophages. *Scale bars* indicate 200 μm. Details of the BFP-MSC and IL13-MSC implant and border are provided, showing activated MHCII^+^eGFP^+^ macrophages (*arrows*) and activated MHCII^+^eGFP^−^ microglia (*arrowheads*). *Scale bars* indicate 50 μm. **e** Cell densities of MHCII^+^eGFP^+^ BM-derived macrophages and MHCII^+^eGFP^−^ microglia within the implant and border of BFP-MSC and IL13-MSC grafts. Values are given as mean + standard deviation. Significant differences (*p* value ≤0.05) are indicated with an *asterisk*. **f** Overview of a BFP-MSC and IL13-MSC graft. *Green*: eGFP fluorescence from graft-infiltrating macrophages. *Red*: Arg1-expressing eGFP^−^ microglia and eGFP^+^ macrophages. *Scale bars* indicate 200 μm. Details of the BFP-MSC and IL13-MSC implant and border are provided, showing alternatively activated Arg1^+^eGFP^+^ macrophages (*arrows*) and Arg1^+^eGFP^−^ microglia (*arrowheads*). *Scale bars* indicate 50 μm. **g** Cell densities of Arg1^+^eGFP^+^ BM-derived macrophages and Arg1^+^eGFP^−^ microglia within the implant and border of BFP-MSC and IL13-MSC grafts. Values are given as mean + standard deviation. Significant differences (*p* value <0.05) are indicated with an asterisk. **h** Overview of a BFP-MSC and IL13-MSC graft. *Green*: eGFP fluorescence from graft-infiltrating macrophages. *Red*: Ym1-expressing eGFP^−^ microglia and eGFP^+^ macrophages. *Scale bars* indicate 200 μm. Details of the BFP-MSC and IL13-MSC implant and border are provided, showing Ym1^+^eGFP^+^ BM-derived macrophages (*arrows*) and Ym1^+^eGFP^−^ microglia (*arrowheads*). *Scale bars* indicate 50 μm

**Fig. 2:**
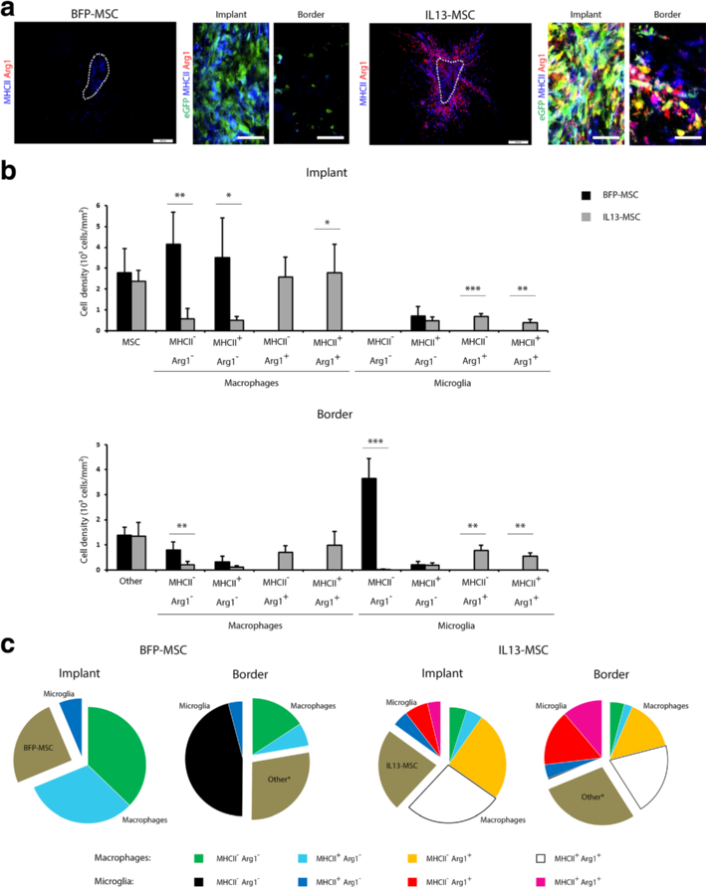
Phenotypic analysis of the different macrophage and microglia populations in BFP-MSC and IL13-MSC grafts in the CNS of eGFP^+^ bone marrow chimaeras. **a** Overview of a BFP-MSC and IL13-MSC graft, showing MHCII-expressing (*blue*) and Arg1-expressing (*red*) microglia and macrophages. *Scale bars* indicate 200 μm. Details of the BFP-MSC and IL13-MSC implant and border are provided, showing graft-infiltrating macrophages (*green*) in combination with Arg1- (*red*) and MHCII-expressing (*blue*) microglia and macrophages. *Scale bars* indicate 50 μm. The following cell populations can be distinguished: eGFP^+^MHCII^+^Arg1^−^ macrophages (*cyan*), eGFP^+^MHCII^−^Arg1^+^ macrophages (*yellow*), eGFP^+^MHCII^+^Arg1^+^ macrophages (*white*), eGFP^+^MHCII^−^Arg1^−^ macrophages (*green*), eGFP^−^MHCII^+^Arg1^−^ microglia (*blue*), eGFP^−^MHCII^−^Arg1^+^ microglia (*red*), eGFP^−^MHCII^+^Arg1^+^ microglia (*magenta*). eGFP^−^MHCII^−^Arg1^−^ microglia (*black background*) cannot be visualised using this setup. *Dotted lines* delineate the actual implants. The border area is an area extending 85 μm from the implant. **b** Cell densities of all different cellular phenotypes characterised within the implant site (*upper graph*) and in the implant border (*lower graph*) of BFP-MSC and IL13-MSC grafts. Values are given as mean + standard deviation. Significant differences are indicated with one (*p* value <0.05), two (*p* value ≤0.01) or three (*p* value ≤0.001) *asterisks*. **c** Pie graphs showing mean representation of each microglia and macrophage phenotype within the BFP-MSC and IL13-MSC graft and border. For the graft site, the fraction of BFP-MSC or IL13-MSC is indicated. For the border, the fraction of other cells is indicated (*neurons, oligodendrocytes and/or astrocytes). The colours of differentially activated microglia and macrophage correspond to those in Fig. 2a

In the first part of this study, we investigated whether microglia, macrophage or astrocyte behaviour was altered upon implantation of IL13-producing MSC (*n* = 5) as compared to implantation of control blue fluorescent protein (BFP)-expressing MSC (*n* = 6) in the CNS of healthy mice. In order to discriminate between microglia and macrophages, BFP-MSC and IL13-MSC were grafted in eGFP^+^ bone marrow chimaeric mice, and MSC grafts were histologically analysed at day 10 post-grafting. As shown by the representative immunofluorescence images in Fig. 1a, a GFAP^+^ astroglial scar delineates both BFP-MSC and IL13-MSC grafts. No noticeable differences were observed at the level of astroglial scarring. Within the MSC graft site, a clear side-by-side presence of grafted BFP-MSC or IL13-MSC (Fig. 1b; arrowheads) and eGFP^+^ BM-derived peripheral macrophages (Fig. 1b; arrows) can be noted. As expected, the majority of BFP-MSC and IL13-MSC graft-invading eGFP^+^ peripheral macrophages reside within the astroglial scar (Fig. 1a). However, upon IL13-MSC grafting, the location of eGFP^+^ BM-derived macrophages seems to be less restricted to the implant site as compared to BFP-MSC grafts (Fig. 1a). In this transplantation model, microglia and macrophages can be distinguished following Iba1 staining (Fig. 1c). As shown, the majority of Iba1^+^ cells within both the BFP-MSC and the IL13-MSC grafts are eGFP^+^ macrophages (Fig. 1c; arrows), whereas the majority of Iba1^+^ cells within the implant border are eGFP^−^ microglia (Fig. 1c; arrowheads). Further quantification indeed confirms that the density of eGFP^+^ macrophages is higher within both the BFP-MSC and the IL13-MSC graft as compared to the graft-surrounding border (both *p* < 0.0001). However, more Iba1^+^ eGFP^+^ macrophages are found in the implant border in the case of IL13-MSC grafts as compared to BFP-MSC grafts (*p* = 0.0121). For graphical representation of these results, see the description of Fig. 2c. In order to further investigate differences between BFP-MSC and IL13-MSC graft-associated microglia and macrophages, we performed additional immunofluorescence stainings to determine the expression of the activation markers MHCII and arginase1 (Arg1). MHCII expression could be detected on eGFP^+^ macrophages and eGFP^−^ microglia within both MSC grafts (Fig. 1d, indicated by arrows and arrowheads, respectively). Furthermore, while MHCII expression was rarely detected on eGFP^+^ macrophages and eGFP^−^ microglia in the border of BFP-MSC grafts (Fig. 1d, indicated by arrows and arrowheads, respectively), its expression is clearly visible on both eGFP^+^ macrophages and eGFP^−^ microglia in the border of IL13-MSC grafts (Fig. 1d, indicated by arrows and arrowheads, respectively). Further quantification provided in Fig. 1e confirms that the highest density of MHCII^+^ cells can be detected among eGFP^+^ macrophages and eGFP^−^ microglia within the BFP- and IL13-MSC grafts, without significant differences between both graft types (Fig. 1e). However, the density of MHCII^+^ eGFP^+^ macrophages and MHCII^+^ eGFP^−^ microglia in the IL13-MSC graft border is significantly higher than in the BFP-MSC graft border (Fig. 1e, both *p* = 0.0346). Next, we found that Arg1 was not expressed on eGFP^+^ macrophages or eGFP^−^ microglia within or surrounding the BFP-MSC grafts (Fig. 1f). However, in the case of IL13-MSC grafts, it can clearly be noted that Arg1^+^ eGFP^+^ macrophages and Arg1^+^ eGFP^−^ microglia can be detected both in and around the graft (Fig. 1f, indicated by arrows and arrowheads, respectively). Further quantification provided in Fig. 1g indeed confirms that Arg1^+^ cells can be detected among eGFP^+^ macrophages and eGFP^−^ microglia within and surrounding the IL13-MSC graft (for all *p* = 0.0233). Another M2 activation marker, Ym1, confirms the observation seen with Arg1 staining, showing only Ym1^+^ eGFP^+^ macrophages and Ym1^+^ eGFP^−^ microglia, in and around the IL13-MSC graft, but not in the BFP-MSC graft (Fig. 1h, indicated by arrows and arrowheads, respectively).

### Detailed characterisation of IL13 induced alterations in the occurrence and spatial distribution of microglia and macrophage phenotypes following MSC grafting

In order to further characterise phenotypic properties of both microglia and macrophages, we performed MHCII/Arg1 double immunofluorescence stainings. As shown in Fig. 2a, double staining for MHCII and Arg1 clearly shows that in the case of IL13-MSC grafting, multiple subtypes of both eGFP^−^ brain-resident microglia and eGFP^+^ peripheral macrophages can be visualised in and around IL13-MSC grafts. Based on these MHCII (in blue) and Arg1 (in red) double stainings, the following populations can be identified: eGFP^+^ MHCII^+^ Arg1^−^ macrophages (cyan), eGFP^+^ MHCII^−^ Arg1^+^ macrophages (yellow), eGFP^+^ MHCII^+^ Arg1^+^ macrophages (white), eGFP^+^ MHCII^−^ Arg1^−^ macrophages (green), eGFP^−^ MHCII^+^ Arg1^−^ microglia (blue), eGFP^−^ MHCII^−^ Arg1^+^ microglia (red) and eGFP^−^ MHCII^+^ Arg1^+^ microglia (magenta). Note that with this staining combination, eGFP^−^ MHCII^−^ Arg1^−^ microglia (black background), which are also present, cannot be visualised in these images. When evaluating the phenotypic properties of control BFP-MSC graft-associated microglia and macrophages, only the following populations can be distinguished: eGFP^+^ MHCII^+^ macrophages (cyan), eGFP^+^ MHCII^−^ macrophages (green) and eGFP^−^ MHCII^+^ microglia (blue). Again, eGFP^−^ MHCII^−^ microglia (black background), which are also present, cannot be visualised on these images. Following quantitative analysis of MHCII^−^ and/or Arg1-expressing cells on eGFP^+^ macrophages and eGFP^−^ microglia, we calculated an approximate biodistribution of the different microglia and macrophage phenotypes observed within and surrounding the BFP- and IL13-MSC grafts (Figs. 2b, c). While Fig. 2b indicates each individual microglia and macrophage subpopulations directly compared between implant site and implant border of BFP-MSC and IL13-MSC grafts, Fig. 2c provides overview pie charts for overall comparison of the differences between BFP-MSC and IL13-MSC grafts. Note the following important similarities and/or differences: (i) macrophages are the major immune cell population within the BFP- and IL13-MSC grafts; (ii) microglia are the major immune cell population surrounding the BFP- and IL13-MSC grafts; (iii) the number of macrophages surrounding the IL13-MSC grafts is significantly higher than the number of macrophages surrounding the BFP-MSC grafts (see above; *p* = 0.0121); (iv) around 50% of macrophages within the BFP-MSC grafts express MHCII, while the majority (80 %) of macrophages within the IL13-MSC grafts express Arg1 with half of them co-expressing MHCII; (v) the small population of microglia within the BFP-MSC grafts expresses MHCII, whereas microglia within the IL13-MSC grafts display a heterogeneous expression of MHCII and/or Arg1; (vi) in the BFP-MSC graft border, only a small population of microglia and macrophages express MHCII; and (vii) in the IL13-MSC graft border, the majority of both microglia and macrophages display a heterogeneous expression of MHCII and/or Arg1. Overall, these results indicate that IL13 secretion by MSC grafts induces a broad spectrum of alternatively activated brain-resident microglia and graft-infiltrating peripheral macrophages, both within and around the MSC graft.

### Non-invasive *T*_2_-weighted MRI reveals a protective effect against CPZ-induced CNS inflammation and demyelination following grafting of IL13-producing MSC

**Fig. 3:**
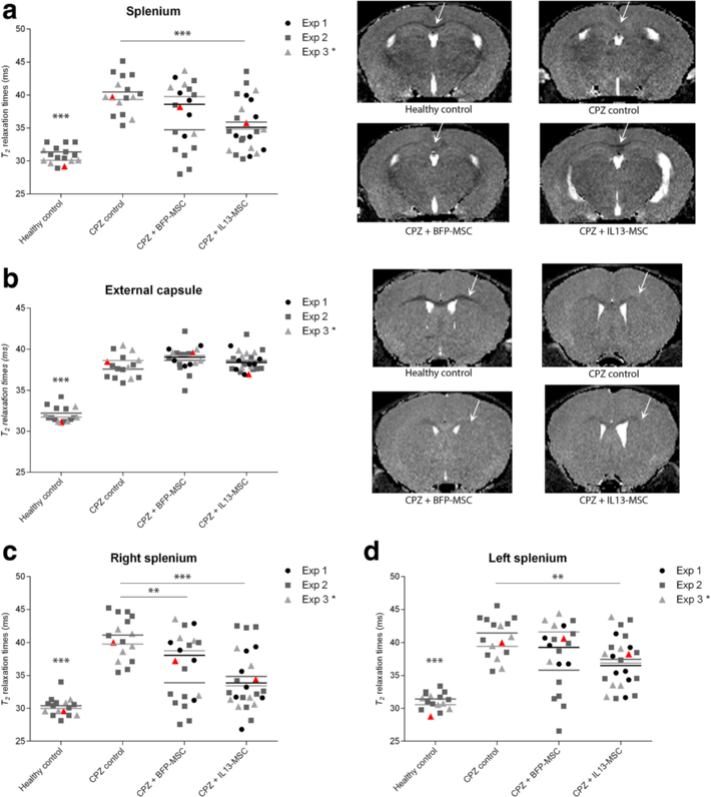
*T*_2_-weighted MRI following injection of BFP-MSC or IL13-MSC on the right side of the splenium in CPZ-treated mice. **a** *Left side*: dot plot showing *T*_2_-relaxation times of the whole *splenium* of the *corpus callosum* (CC) in healthy control mice (*n* = 16), in CPZ-treated mice (CPZ control, *n* = 17), in BFP-MSC grafted CPZ-treated mice (CPZ + BFP-MSC, *n* = 20) and in IL13-MSC grafted CPZ-treated mice (CPZ + IL13-MSC, *n* = 24). Each data point corresponds to the value obtained from an individual mouse of one of the three independent experiments (described in Table 1). The mean of each independent experiment is represented by a horizontal line. *Right side*: representative MRI image demonstrating intensity alterations in the *splenium* for each group (area indicated by *arrows*). **b** *Left side*: dot plot showing *T*_2_-relaxation times of the whole external capsule (EC) for each experimental group. The mean of each independent experiment is represented by a horizontal line. *Right side*: representative MRI image demonstrating intensity alterations in the EC for each group (area indicated by the *arrows*). **c** Dot plot showing *T*_2_-relaxation times of the right side of the *splenium* for each experimental group. The mean of each independent experiment is represented by a horizontal line. **d** Dot plot showing *T*_2_-relaxation times of the left side of the *splenium* for each experimental group. The mean of each independent experiment is represented by a horizontal line. For all dot plots, significant differences versus the CPZ control group are indicated with one (*p* value <0.05), two (*p* value ≤0.01) or three (*p* value ≤ 0.001) asterisks. Data points indicated in *red* on (**a**, **b**, **c** and **d**) correspond with the MRI images provided in (**a** and **b**) and the histological images shown in Fig. 4a

Next, we investigated whether grafting of IL13-producing MSC, and the subsequent secretion of IL13 and/or the appearance of M2 polarised microglia and macrophages, could influence neuroinflammatory responses. For this, we performed three independent experiments of which the details of employed mouse strains (wt C57BL/6 or C57BL/6 CX_3_CR1^eGFP/+^ CCR2^RFP/+^ mice), grafted cell types (BFP-MSC, IL13-MSC or IL13/BFP-MSC) and size of the different experimental groups (healthy control, CPZ control, CPZ + BFP-MSC and CPZ + IL13-MSC) are provided in Table 1. Experimentally, 8-week-old mice received a BFP-MSC or an IL13-MSC graft at the right side of the *splenium* of the *corpus callosum* followed by a 4-week CPZ-supplemented diet. Healthy control mice were kept on normal rodent diet for 4 weeks, while CPZ control mice received a 4-week CPZ-supplemented diet without undergoing surgical intervention. At the age of 12 weeks, *T*_2_-weighted MRI was applied to non-invasively measure CPZ-induced inflammation and demyelination in the *splenium* and the *external capsule* (EC, internal control region) [30]. As no significant differences in the calculated *T*_2_ relaxation times for the *splenium* and the EC were found between wt C57BL/6 (used in experiments 1 and 2) and C57BL/6 CX_3_CR1^eGFP/+^ CCR2^RFP/+^ mice (used in experiment 3), under both healthy conditions as well as under CPZ treatment, data from all three experiments were used to increase power in further statistical analyses (Fig. 3a, b, experiment 2 versus experiment 3, healthy control and CPZ control). When comparing the calculated *T*_2_ relaxation time for the whole *splenium* (both right and left sides of the midline) and the whole EC (both right and left sides) from healthy control mice and non-injected CPZ-treated mice (CPZ control), it is clear that CPZ treatment causes a significant increase of the *T*_2_ relaxation time in both brain regions (Fig. 3a, b, for both *p* < 0.0001). This can also be noted on the representative MRI images provided where hyperintensity regions were seen in the *splenium* and EC of CPZ control mice, a finding not observed in healthy control mice (Fig. 3a, b). When comparing the *T*_2_ relaxation times of the whole *splenium* in BFP-MSC with IL13-MSC grafted mice, data indicate that *T*_2_ relaxation times of IL13-MSC grafted mice, but not of BFP-MSC grafted mice, deviate significantly less from healthy control values compared to those obtained from CPZ-treated mice (Fig. 3a, CPZ control versus CPZ + IL13-MSC, *p* = 0.0007). This is indicative of reduced inflammation and demyelination in the *splenium* of IL13-MSC grafted mice. The latter can also be noted on the representative MRI images provided in Fig. 3a, where the *splenium* in IL13-MSC grafted mice displays less hyperintensity as compared to CPZ control mice. The observation that *T*_2_ relaxation times of the EC remained high and were not significantly different from those obtained in CPZ control mice (Fig. 3b) indicates that the observed protective effect following grafting of IL13-MSC is due to the performed cell therapeutic intervention and does not result from model failure and/or model variation. Of note, we observed in some of the BFP-MSC grafted mice that this procedure might have led to neuroprotection. However, as this was only observed in part of the mice from one out of three MRI experiments, the results convince that IL13-MSC grafting is superior to control BFP-MSC grafting in establishing protection against inflammation and demyelination in the whole *splenium.* Further analyses over the right and left sides of the *splenium* by *T*_2_-weighted MRI indicates that the protective effect of grafting IL13-expressing MSC is of higher significant importance on the right side of the *splenium* (*p* < 0.0001) as compared to the left side of the *splenium* (*p* = 0.01) (Fig. 3c, d).

**Table 1:** Experimental outline of the three comparative cell grafting experiments performed in the cuprizone (CPZ) mouse model

Exp.	Mouse strain	Cell graft	Healthy control	CPZ control	CPZ + BFP-MSC	CPZ + IL13-MSC
1	C57BL/6 wt	BFP-MSC IL13-MSC	–	–	*n* = 5	*n* = 7
2	C57BL/6 wt	BFP-MSC IL13-MSC	*n* = 10	*n* = 10	*n* = 10	*n* = 10
3	C57BL/6 CX_3_CR1^eGFP/+^CCR2^RFP/+^ tg	BFP-MSC IL13/BFP-MSC^a^	*n* = 6	*n* = 6	*n* = 5	*n* = 7
Total number of mice			*n* = 16	*n* = 16	*n* = 20	*n* = 24

*Exp* experiment, *wt* wild type, *tg* transgenic, *MSC* mesenchymal stem cell, *BFP-MSC* blue fluorescent protein-expressing MSC, *IL13-MSC* interleukin 13-expressing MSC, *IL13/BFP-MSC* IL13 + BFP-expressing MSC, *n* number of mice

^a^To avoid confusion, both IL13-producing cell lines, IL13- and IL13/BFP-MSC, will be referred to as IL13-MSC further in text

### Histological evaluation confirms protection against CPZ-induced CNS inflammation and demyelination by IL13-secreting MSC

**Fig. 4:**
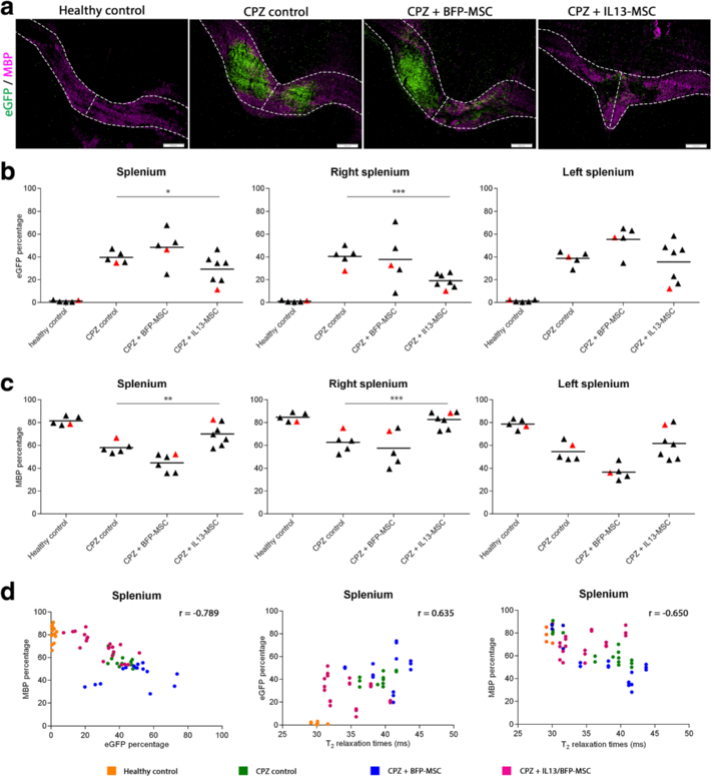
Quantification of microglia and myelin coverage following injection of BFP-MSC or IL13-MSC on the right side of the splenium in CPZ-treated mice. **a** Representative overview images of the *splenium* of the *corpus callosum* with CX_3_CR1^+^ microglia (*green*) and MBP^+^ myelin (*purple*) in control, CPZ-treated mice and CPZ-treated mice with BFP-MSC grafts or IL13-MSC grafts. The areas which were analysed are delineated by a *dotted line. Scale bar* indicates 200 μm. **b** Dot plots showing the coverage percentage of eGFP in the whole *splenium* (*left graph*), the right *splenium* (*middle graph*) and the left *splenium* (*right graph*) for each experimental group. **c** Dot plots showing the coverage percentage of MBP in the whole *splenium* (*left graph*), the right *splenium* (*middle graph*) and the left *splenium* (*right graph*) for each experimental group. The mean of each independent experiment is represented by a *horizontal line*. Significant differences are indicated with one (*p* value <0.05), two (*p* value ≤ 0.01) or three (*p* value ≤ 0.001) *asterisks*. **d** Dot plots showing the correlation between eGFP and MBP coverage percentage (*left graph*), eGFP coverage percentage and *T*_2_ relaxation times (*middle graph*), MBP coverage percentage and *T*_2_ relaxation times (*right graph*) in the *splenium* for each experimental group. Spearman’s correlation coefficient (*r*) is presented on each graph. Data points indicated in *red* on (b and c) correspond with the representative MRI images provided in Fig. 3a, b and with the representative histological images shown in (**a**)

Next, we performed immunofluorescent stainings for myelin basic protein (MBP) on C57BL/6 CX_3_CR1^eGFP/+^ CCR2^RFP/+^ mice for which MRI data were obtained (MRI experiment 3, *n* = 5 for healthy control, *n* = 5 for CPZ control, *n* = 5 for CPZ + BFP-MSC and *n* = 7 for CPZ + IL13-MSC), and assessed both the degree of CX_3_CR1^eGFP/+^ microglia activity (as determined by the percentage of eGFP coverage) and the degree of myelination (as determined by the percentage of MBP coverage) in the whole *splenium*, the right side of the *splenium* and the left side of the *splenium* (Fig. 4). As shown by the representative images in Fig. 4a, it is clear that the *splenium* becomes highly invaded by CX_3_CR1^eGFP/+^ microglia following CPZ treatment. As expected from the above-mentioned MRI data, representative images of IL13-MSC grafted mice show a decrease in CX_3_CR1^eGFP/+^ microglia density in the *splenium*, which was not observed in BFP-MSC grafted mice. In agreement with the aforementioned MRI data, further quantitative analysis of microglia activity (Fig. 4b) within the whole, the right and the left side of the *splenium* confirms a significant decrease following grafting of IL13-MSC over the whole *splenium* (*p* = 0.02), and more specifically at the right side (*p* < 0.0001) of the *splenium*, as compared to non-grafted CPZ control mice. In contrast, no overall significant decrease in microglia activity could be detected following grafting of BFP-MSC in the *splenium*, as compared to non-grafted CPZ control mice. Concerning the degree of myelination, it is clear from the representative images provided in Fig. 4a that severe demyelination can be observed at the site of CX_3_CR1^+^ microglia accumulation upon CPZ administration. Moreover, as expected from the MRI and representative images of IL13-MSC grafted mice, protection against demyelination of the *splenium* is suggested. Further quantitative analysis of myelination (Fig. 4c) within the whole, the right and the left side of the *splenium* confirms a significant protection against demyelination following grafting of IL13-MSC over the whole *splenium* (*p* = 0.0011) and the right side of the *splenium* (*p* < 0.0001), as compared to non-grafted CPZ control mice. Again, no overall significant protection against demyelination could be detected following grafting of BFP-MSC in the *splenium*, as compared to non-grafted CPZ control mice.

### *T*_2_-weighted MRI results strongly correlate with histology data

As we have obtained a large set of corresponding MRI and histology data, we investigated if non-invasive *T*_2_-weighted MRI observations can be used as a reliable surrogate analysis to assess inflammation and demyelination in the CPZ mouse model. When histologically evaluating CX_3_CR1^eGFP/+^ microglia density and MBP myelination, a very strong negative correlation (*r* = −0.789; 95 % confidence interval [−0.91; −0.50]) was found between the microglia density and myelination over the whole *splenium* (Fig. 4d, left graph). When evaluating *T*_2_-weighted MRI data with histological data showing CX_3_CR1^eGFP/+^ microglia density, a strong positive correlation (*r* = 0.635; 95 % confidence interval [0.22; 0.86]) was found between the increase in microglia density and the increase of *T*_2_ relaxation time over the whole *splenium* (Fig. 4d, middle graph). When evaluating *T*_2_-weighted MRI data with histological data showing MBP myelin density, a strong negative correlation (*r* = -0.650; 95 % confidence interval [−0.84; −0.29]) was found between the decrease in MBP density and the increase of *T*_2_ relaxation time over the whole *splenium* (Fig. 4d, right graph). Based on these correlation analyses, we are confident that the observed *T*_2_-weighted MRI images reflect the obtained histological data.

### Differential behaviour of microglia and macrophages in IL13-expressing MSC graft-mediated modulation of CPZ-induced neuroinflammation and demyelination

**Fig. 5:**
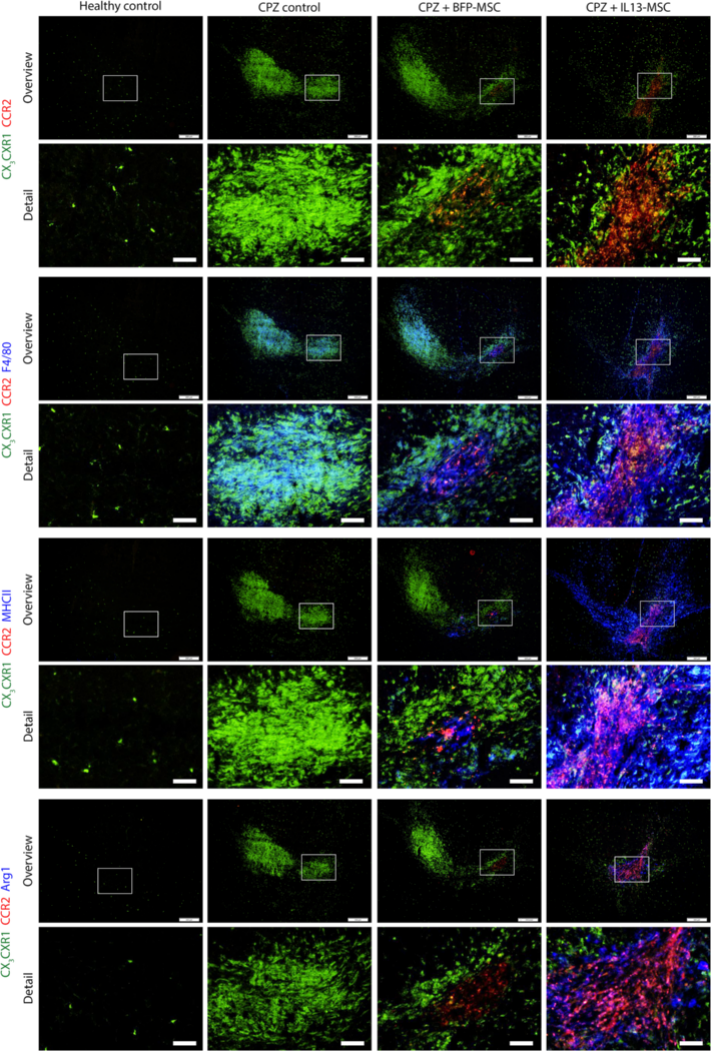
Representative images illustrating multiple phenotypes of microglia and macrophages in the control and experimental groups. Representative overview images (*odd rows*, *scale bar* indicates 200 μm) of the *splenium* of the *corpus callosum* (CC) in control mice (*first column*), CPZ-treated mice (*second column*) and CPZ-treated mice with BFP-MSC grafts (*third column*) or IL13-MSC grafts (*fourth column*). Representative detail images (*even rows*, *scale bar* indicates 50 μm) from the BFP-MSC or IL13-MSC graft site or from the corresponding areas in healthy control or CPZ control mice. *First* and *second rows*: in *green* direct eGFP fluorescence from CX3CR1^+/eGFP^ brain-resident microglia and in *red* direct RFP fluorescence from peripheral CCR2^+/RFP^ macrophages. *Third* and *fourth* rows: additionally in *blue* immunofluorescent staining for F4/80 marking activated eGFP^+^ brain-resident microglia and RFP^+^ peripheral macrophages. *Fifth* and *sixth* rows: additionally in blue immunofluorescent staining for MHCII marking activated eGFP^+^ brain-resident microglia and RFP^+^ peripheral macrophages. *Seventh* and *eighth* rows: additionally in *blue* immunofluorescent staining for Arg1 marking alternatively activated eGFP^+^ brain-resident microglia and RFP^+^ peripheral macrophages

Next, we took advantage of the CX_3_CR1^eGFP/+^ CCR2^RFP/+^ mouse model to unravel the contribution and/or phenotypic properties of microglia and macrophages during CPZ-induced inflammation and the effect of IL13-expressing MSC grafts. For this, further histological analyses were performed on C57BL/6 CX_3_CR1^eGFP/+^ CCR2^RFP/+^ mice from MRI experiment 3, of which representative overview and detail images of the *splenium* are provided in Fig. 5. For each of the experimental conditions, images discriminate CX_3_CR1^+^ microglia (eGFP fluorescence) from CCR2^+^ infiltrating peripheral macrophages (RFP fluorescence). Additional activation markers, including F4/80, MHCII and Arg1, are shown in blue (Fig. 5). While in healthy control mice, only small numbers of CX_3_CR1^eGFP/+^ microglia are present within the *splenium*, and following CPZ treatment, the *splenium* becomes highly invaded by CX_3_CR1^eGFP/+^ microglia without noticeable contribution of CCR2^RFP/+^ peripheral macrophages. Representative images of IL13-MSC grafted mice, but not BFP-grafted mice, show a remarkable decrease in CX_3_CR1^eGFP/+^ microglia density in the whole *splenium* (Fig. 5, first and second rows, direct CX_3_CR1-eGFP fluorescence and direct CCR-RFP fluorescence, quantification provided in Fig. 4b). In agreement with our previously published data on the spatial distribution of microglia and BM-derived macrophages following MSC transplantation in the CNS, and with the data presented in the first part of this manuscript, both MSC graft types in CPZ-treated CX_3_CR1^eGFP/+^ CCR2^RFP/+^ mice are invaded by CCR2^RFP/+^ peripheral macrophages and surrounded by CX_3_CR1^eGFP/+^ brain-resident microglia (Fig. 5, second row, direct CX_3_CR1-eGFP fluorescence and direct CCR2-RFP fluorescence) [13]. Clearly, following MSC grafting in the CPZ model, we can thus distinguish two separate inflammatory responses: on one hand, CPZ-induced CX_3_CR1^eGFP/+^ microglia activation in the *splenium* which can be modulated following grafting of IL13-expressing MSC; on the other hand, MSC graft-associated CX_3_CR1^eGFP/+^ microglia and CCR2^RFP/+^ peripheral macrophage activation. Upon further evaluation of the activation status of microglia and macrophages, by staining for F4/80 in each of the experimental groups, it is clear that CPZ pathology-associated CX_3_CR1^eGFP/+^ microglia, as well as MSC graft-associated CX_3_CR1^eGFP/+^ microglia and CCR2^RFP/+^ peripheral macrophages, express F4/80 (Fig. 5, third and fourth rows, blue staining), while expression of this activation marker is absent on steady-state resting microglia in healthy brain tissue. MHCII expression is mainly restricted to CCR2^RFP/+^ peripheral macrophages invading the BFP-MSC graft, whereas MHCII is expressed on both IL13-MSC graft-infiltrating CCR2^RFP/+^ peripheral macrophages and on graft-surrounding CCR2^RFP/+^ macrophages and CX_3_CR1^eGFP/+^ microglia (Fig. 5, fifth and sixth rows, blue staining). Also note that no MHCII expression was detected on CPZ pathology-associated CX_3_CR1^eGFP/+^ microglia (Fig. 5, fifth and sixth rows). Finally, staining for Arg1 (Fig. 5, seventh and eighth rows, blue staining) can only be detected on IL13-MSC graft-infiltrating and graft-surrounding CCR2^RFP/+^ peripheral macrophages and CX_3_CR1^eGFP/+^ microglia. These results indicate that IL13 produced by grafted MSC is able to (i) trigger alternative activation of MSC graft-associated microglia and macrophages also in the diseased brain and (ii) diminish CPZ-induced inflammation and demyelination by its direct action or in combination with the action of alternatively activated macrophages/microglia.

### Non-invasive *T*_2_-weighted MRI reveals that two injections of IL13-expressing MSC results in superior protection against CNS inflammation and demyelination

**Fig. 6:**
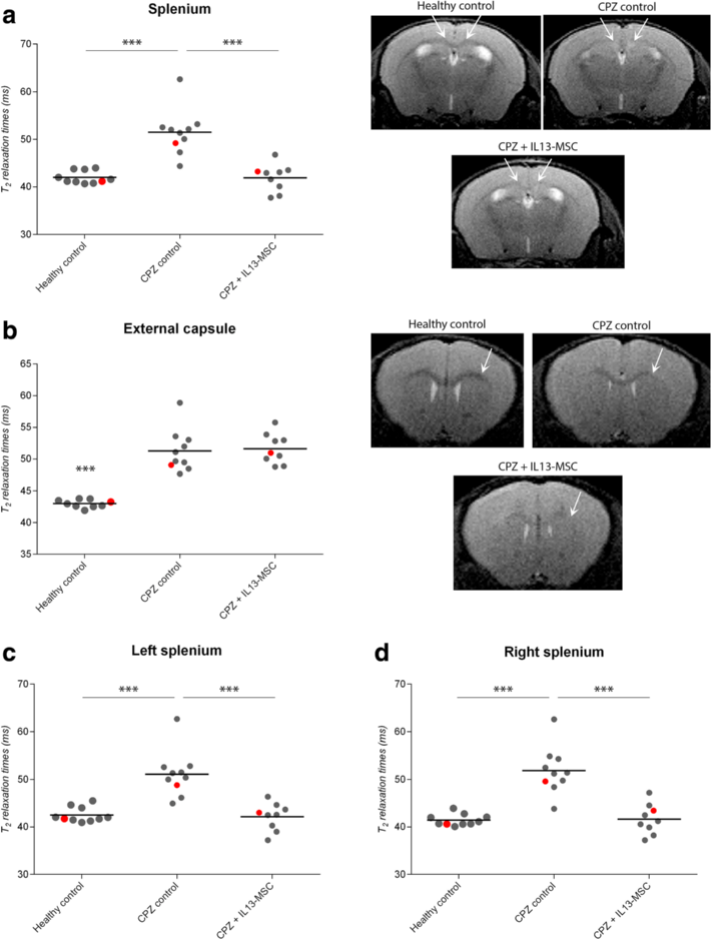
*T*_2_-weighted MRI analysis following injection of IL13-MSC on the right and the left side of the splenium in CPZ-treated mice. **a** Left side: dot plot showing *T*_2_-relaxation times of the whole *splenium* of the *corpus callosum* (CC) in healthy control mice (*n* = 10), in CPZ-treated mice (CPZ control, *n* = 10) and in IL13-MSC grafted CPZ-treated mice (CPZ + IL13-MSC, *n* = 10). *Right side*: representative MRI image demonstrating intensity alterations in the *splenium* for each group (area indicated by *arrows*). **b** *Left side*: dot plot showing *T*_2_-relaxation times of the whole external capsule (EC) for each experimental group. *Right side*: representative MRI image demonstrating intensity alterations in the EC for each group (area indicated by the *arrows*). **c** Dot plot showing *T*_2_-relaxation times of the right side of the *splenium* for each experimental group. **d** Dot plot showing *T*_2_-relaxation times of the left side of the *splenium* for each experimental group. Significant differences versus the CPZ control group are indicated with *three asterisks* (*p* value ≤ 0.001). Data points indicated in *red* on (**a**, **b**, **c** and **d**) correspond with the MRI images provided in (**a** and **b**) and with the histological images shown in Fig. 7a and b

Given the observation that grafting of IL13-MSC on the right side of the splenium confers histopathological protection only on the right side of the splenium, we next investigated whether grafting of IL13-MSC on both the left and right sides of the splenium could induce a stronger and more widespread effect. In this study, 8-week-old wild-type C57BL/6 mice were divided over three experimental groups: a healthy control group (*n* = 10) which received a normal rodent diet for 4 weeks, a CPZ control group (*n* = 10) which received 4 weeks of CPZ-supplemented diet without any injections and an IL13-MSC group (*n* = 10) which received 4 weeks of CPZ-supplemented diet and IL13-MSC injections at both the right and left sides of the splenium. First, MRI was applied to non-invasively evaluate CPZ-induced inflammation and demyelination in the splenium and the EC. When evaluating *T*_2_ relaxation times over the whole splenium, it is clear that grafting of IL13-MSC highly significantly (*p* < 0.0001) prevents deviation from normal *T*_2_ relaxation times upon cuprizone administration (Fig. 6a). Again, the observed neuroprotection in the splenium is specific to the IL13-MSC grafting procedure as *T*_2_ relaxation times in the EC were equally well affected by the cuprizone diet in both the CPZ control group and the IL13-MSC group (Fig. 6b, for both *p* < 0.0001). Most interestingly, using this experimental setup we are able to confirm that grafting of IL13-MSC both on the right and left sides of the splenium is superior to a single graft on the right side. When evaluating *T*_2_ relaxation times separately on the left and right sides of the splenium, it is clear that the observed protection is now equally well distributed over both sides of the splenium (Fig. 6c, d; for all *p* ≤ 0.0001).

### Histological evaluation confirms that reduced demyelination following grafting of IL13-MSC is associated with oligodendrocyte survival in the splenium of CPZ-treated mice

**Fig. 7:**
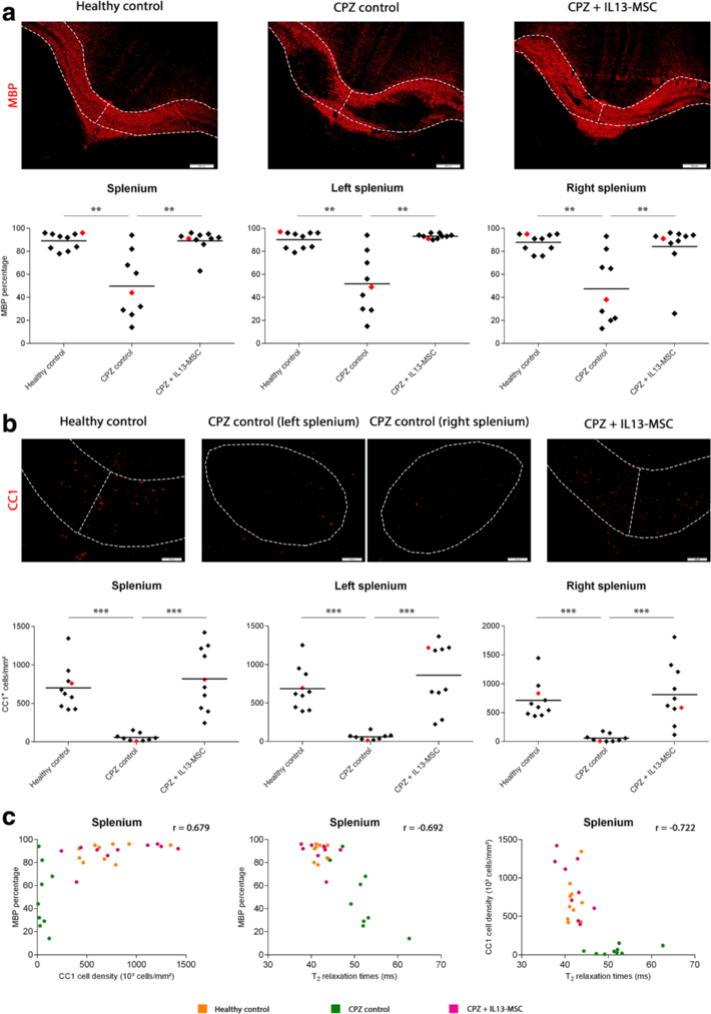
Quantification of myelin coverage and oligodendrocyte density following injection of IL13-MSC on the right and left sides of the splenium in CPZ-treated mice. **a** *Upper row*: representative overview images of the *splenium* of the *corpus callosum* stained for MBP (*red*) in control, CPZ-treated mice and CPZ-treated mice with IL13-MSC grafts. The areas which were analysed are delineated by a *dotted line. Scale bar* indicates 200 μm. *Lower row*: dot plots showing the coverage percentage of MBP over the whole *splenium* (*left graph*), the left side of the *splenium* (*middle graph*) and the right side of the *splenium* (*right graph*) for each experimental group. The mean of each group is represented by a *horizontal line*. Significant differences are indicated with two (*p* value ≤0.01) or three (*p* value ≤0.001) *asterisks*. **b** *Upper row*: representative overview images of the *splenium* of the *corpus callosum* stained for CC1^+^ oligodendrocytes (*red*) in control, CPZ-treated mice and CPZ-treated mice with IL13-MSC grafts. The areas which were analysed are delineated by a *dotted line. Scale bar* indicates 100 μm. *Lower row*: dot plots showing the cell density (cells/mm^2^) of CC1^+^ oligodendrocytes over the whole *splenium* (*left graph*), the left side of the *splenium* (*middle graph*) and the right side of the *splenium* (*right graph*) for each experimental group. The mean of each group is represented by a *horizontal line*. Significant differences are indicated with *two* (*p* value ≤0.01) or *three* (*p* value ≤0.001) *asterisks*. **c** Dot plots showing the correlation between MBP coverage percentage and CC1^+^ oligodendrocyte cell density (*left graph*), MBP coverage percentage and *T*_2_ relaxation times (middle graph), CC1^+^ oligodendrocyte cell density and *T*_2_ relaxation times (*right graph*) in the whole *splenium* for each experimental group. Spearman’s correlation coefficient (r) is presented on each graph. Data points indicated in *red* on (**a** and **b**) correspond with the representative MRI images provided in Fig. 6a, b and with the representative histological images shown in (**a** and **b**)

In agreement with our MRI data, histological analysis first confirmed that grafting of IL-13 MSC at both sides of the splenium prevents demyelination over the whole splenium, both at the right and left sides (Fig. 7a; IL13-MSC versus CPZ control, *p* = 0.0045, *p* = 0.0025 and *p* = 0.0062, respectively). These results thus further confirm our hypothesis that grafting of IL13-MSC on both the left and right sides of the splenium could induce a stronger and more widespread effect as compared to IL13-MSC grafting only at the right side (Figs. 3 and 4). Next, we aimed to investigate whether the presence of myelin in the IL13-MSC group could be related to the actual survival/presence of oligodendrocytes. For this, an additional immunostaining for CC1 was performed and the density of CC1^+^ oligodendrocytes was determined within the whole, left side and right side of the splenium (Fig. 7b). While CC1^+^ oligodendrocyte death is highly prominent in the CPZ control group, their survival is highly significant in the whole, left side and right side of the splenium following IL13-MSC grafting at both the left and right sides of the splenium (IL13-MSC versus CPZ control, for all regions *p* ≤ 0.0001). These results thus indicate that IL13-mediated modulation of CPZ-induced inflammatory responses can interfere with CPZ/inflammation-induced oligodendrocyte death and subsequent demyelination. Finally, having obtained this large dataset, Spearman correlation analysis showed a strong positive correlation (*r* = 0.679; 95% confidence interval [0.40; 0.86]) over the whole splenium between MBP coverage percentage and CC1^+^ cell density (Fig. 7c, left graph). In addition, a strong negative correlation (*r* = -0.692; 95 % confidence interval [−0.87; −0.38]) was found over the whole splenium between MBP coverage percentage and *T*_2_ relaxation times (Fig. 7c, middle graph). Similarly, a strong negative correlation (*r* = −0.722; 95 % confidence interval [−0.84; −0.47]) could be defined over the whole splenium between CC1^+^ cell density and *T*_2_ relaxation times (Fig. 7c, right graph). These correlation analyses again demonstrate that the MRI analyses reflect the obtained histological data.

## Discussion

In the first part of this study, we evaluated the use of autologous MSC grafts in the CNS as a delivery vehicle for the immune modulating cytokine IL13. With regard to our choice to use MSC as a delivery vehicle, we previously reported extensively on the cellular remodelling of mesenchymal (stem) cell grafts in the CNS of healthy mice. From these studies, it is now well-accepted that this intervention is a highly complex interplay between hypoxia-induced apoptosis of the grafted cells, neutrophil invasion, neo-angiogenesis, microglia/macrophage recruitment, astrogliosis and eventually survival of a significant (5–30%) number of grafted cells [11–15, 31]. In previous studies, we also reported that graft-recognising microglia/macrophages display a pro-inflammatory phenotype as shown by the temporary expression of CD11b and MHCII. It should be noted however that, at the time of these studies, we were unable to experimentally distinguish brain-resident microglia from CNS-invading macrophages [12]. In this new study, we highlight the potential of IL13 secretion by grafted MSC to induce alternative activation both in MSC graft-surrounding microglia and in MSC graft-infiltrating macrophages in vivo, as indicated by the expression of Arg1 and Ym1 (Fig. 1f–h). Moreover, from our study it is also clear that IL13 priming of macrophages and microglia in vivo does not result in a single clearly distinguishable cell population (Fig. 2). We may speculate that each of the microglia or macrophage phenotypes described, either single positive for MHCII, single positive for Arg1 or double positive, may exert a different effector function; however, this remains to be further investigated on the single cell level. Thus far, apart from the induction of markers associated with alternative activation, we currently do not know what influence IL13 and/or M2 polarised microglia/macrophages display on the in vivo survival of autologous MSC grafts and/or their in vivo remodelling. While our first observations do not indicate a major difference between autologous MSC grafts with and without genetic engineering to secrete IL13 (Fig. 1a–e), we recently reported that transplantation of allogeneic MSC genetically engineered to secrete IL13, with subsequent induction of Arg1, Ym1 and Fizz1 expression in MSC graft-recognising microglia and/or macrophages, leads to highly reduced downstream effector function in terms of (i) direct in vivo recognition and elimination of allogeneic cellular grafts and (ii) in vivo induction of allogeneic T cell immune responses [32]. Although the process of direct and indirect allorecognition does not occur with autologous (syngeneic) cellular grafts, we may speculate that IL13 can have a beneficial effect on long-term engraftment of autologous MSC grafts in the CNS; however, this needs further investigation.

In the second part of our manuscript, we provide a thorough analysis of MSC graft-mediated IL13 delivery as an effective approach to modulate pathology-associated immune responses in the CNS of mice. We here highlight that IL13 production by grafted MSC, potentially aided by the appearance of alternatively activated microglia and macrophages at the MSC graft site, prevents microgliosis, oligodendrocyte death and demyelination in the CPZ mouse model (Figs. 4 and 7). Note that the therapeutic approach presented here, i.e. intracerebral grafting of IL13-producing MSC, is significantly different from several previously published reports regarding the therapeutic use of non-engineered MSC in the CPZ mouse model. A study by Nessler and colleagues was unable to report any clinical benefit of both intravenously or intranasally administered MSC [33], most likely due to limited cell migration to the CNS. In addition, our own preceding work on cell grafting in the CPZ model at the peak of inflammation and demyelination was also unable to demonstrate any contribution of mesenchymal, neural or haematopoietic cells to remyelination [14]. The latter observation was recently confirmed by a study performed by Salinas Tejedor et al. showing that MSC injected at the onset or the peak of oligodendrocyte proliferation during cuprizone-induced demyelination do not exert beneficial effects on remyelination [34]. Although all these studies question the use of MSC to exert direct neuroprotective effects in the CPZ model, we here provide an alternative approach in which we demonstrate that autologous MSC are well-suited as transplantable carrier cells for the direct delivery of a therapeutic protein to the injured CNS, as proven in the current study for the M2-polarising cytokine IL13. However, currently still open for debate is the actual working mechanism for the observed neuroprotective character of MSC-mediated IL13 delivery in the CNS. Based on our current knowledge, and in agreement with several literature reports, we propose the following working hypothesis. Previously, it has been described by Yang et al. that IL13 can directly induce apoptosis in activated microglia [35]. It is not unlikely that this feature of IL13 highly contributes to the reduced microgliosis, and subsequent reduced oligodendrocyte death and demyelination, observed in this study. Although we prefer to support this first hypothesis, M2 polarised macrophages/microglia, which are effectively introduced in the CNS by means of local transplantation of IL13-producing MSC, may also indirectly contribute to the observed protection against demyelination. In this context, it has previously been reported that M2 polarised macrophages are able to promote oligodendrocyte differentiation and enhance remyelination [36]. However, using the current experimental setup we cannot make a clear distinction between protection against inflammation-induced oligodendrocyte death and/or already occurred oligodendrocyte regeneration. Likewise, given the intrinsic toxic nature of CPZ to oligodendrocyte metabolism [17], it remains to be elucidated whether the observed myelin is fully functional on the structural and functional level. For this, electron microscopy [37, 38] and more advanced MRI studies [26, 27, 39] are planned in our future experiments. Nevertheless, as the field of CNS immunomodulation is highly complex and continuously evolving, a highly complex interplay between both proposed mechanisms may not be not unlikely.

Finally, we would like to elaborate a little on the proposed experimental strategies used in this study. First, we applied non-invasive *T*_2_-weighted MRI (Figs. 3 and 6) and correlated these findings with post-mortem histological analyses (Figs. 4 and 7). Although MRI analyses are not novel for the CPZ mouse model, we here demonstrate that therapeutic interventions in the CPZ mouse model can successfully be detected by means of *T*_2_-weighted MRI. Therefore, longitudinal MRI studies of the CC in the CPZ mouse model may/should become highly complementary to single point post-mortem histological analyses when applying therapeutic interventions, as previously suggested by us [39]. Second, our MSC implantation approach deserves some special attention. The presented studies here suggest that multiple injections of IL13-MSC are superior over a single injection (Figs. 3, 4, and 5 versus Figs. 6 and 7). Although this approach solves the issue of the limited local action of IL13-MSC grafting, one should take caution with multiple cellular injections in the CNS, especially when considering human applications in future. However, as to date the exact working mechanism of cellular therapies in the CNS is largely unresolved, in addition to the direct action of IL13, we previously already suggested that MSC graft-induced inflammatory responses (in this study the introduction of M2 polarised macrophages into the CNS) may lie at the basis of observed neuroprotective and/or regenerative responses [31].

## Conclusions

With this study, we propose a novel approach for effective modulation of detrimental in vivo inflammatory responses in the CNS, where IL13 production by grafted MSC and potentially aided by the appearance of alternatively activated microglia and macrophages at the MSC graft site, induces microglial quiescence and subsequent protection against oligodendrocyte death and demyelination in the CPZ mouse model for CNS inflammation and demyelination. In this context, we believe our experimental approach to be worthy of further evaluation in clinically relevant mouse models of neurotrauma displaying a gradual increase in inflammatory responses, e.g. models of spinal cord injury or stroke.

## Abbreviations

BFP: Blue fluorescent protein
BM: Bone marrow
CC: Corpus callosum
CEM: Complete expansion medium
CNS: Central nervous system
CPMG: Carr-Purcell-Meiboom-Gill
CPZ: Cuprizone
EC: External capsule
eGFP: Enhanced green fluorescent protein
FBS: Fetal bovine serum
FOV: Field of view
GEE: Generalised estimating equations
HS: Horse serum
IL13: Interleukin 13
IMDM: Iscove’s modified Dulbecco’s medium
MBP: Myelin basic protein
MS: Multiple sclerosis
MSC: Mesenchymal stem cell
MSME: Multi-slice multi-echo
NA: Number of averages
NS: Number of slices
NSC: Neural stem cell
PBS: Phosphate buffered saline
PFA: Paraformaldehyde
RFP: Red fluorescent protein
ROI: Regions of interest
TE: Echo times
TR: Repetition time
UA: University of Antwerp

## References

[1] Benakis C, Garcia-Bonilla L, Iadecola C, Anrather J (2014). The role of microglia and myeloid immune cells in acute cerebral ischemia. Front Cell Neurosci.

[2] Morganti JM, Jopson TD, Liu S, Riparip LK, Guandique CK, Gupta N, Ferguson AR, Rosi S (2015). CCR2 antagonism alters brain macrophage polarization and ameliorates cognitive dysfunction induced by traumatic brain injury. J Neurosci.

[3] Yamasaki R, Lu H, Butovsky O, Ohno N, Rietsch AM, Cialic R, Wu PM, Doykan CE, Lin J, Cotleur AC (2014). Differential roles of microglia and monocytes in the inflamed central nervous system. J Exp Med.

[4] Zhou X, He X, Ren Y (2014). Function of microglia and macrophages in secondary damage after spinal cord injury. Neural Regen Res.

[5] Shechter R, Schwartz M (2013). Harnessing monocyte-derived macrophages to control central nervous system pathologies: no longer ‘if’ but ‘how’. J Pathol.

[6] Murray PJ, Allen JE, Biswas SK, Fisher EA, Gilroy DW, Goerdt S, Gordon S, Hamilton JA, Ivashkiv LB, Lawrence T (2014). Macrophage activation and polarization: nomenclature and experimental guidelines. Immunity.

[7] Van Dyken SJ, Locksley RM (2013). Interleukin-4- and interleukin-13-mediated alternatively activated macrophages: roles in homeostasis and disease. Annu Rev Immunol.

[8] Ibraheem D, Elaissari A, Fessi H (2014). Gene therapy and DNA delivery systems. Int J Pharm.

[9] De Vocht N, Praet J, Reekmans K, Le Blon D, Hoornaert C, Daans J, Berneman Z, Van der Linden A, Ponsaerts P (2013). Tackling the physiological barriers for successful mesenchymal stem cell transplantation into the central nervous system. Stem Cell Res Ther.

[10] Bergwerf I, Tambuyzer B, De Vocht N, Reekmans K, Praet J, Daans J, Chatterjee S, Pauwels P, Van der Linden A, Berneman ZN, Ponsaerts P (2011). Recognition of cellular implants by the brain’s innate immune system. Immunol Cell Biol.

[11] Costa R, Bergwerf I, Santermans E, De Vocht N, Praet J, Daans J, Le Blon D, Hoornaert C, Reekmans K, Hens N (2015). Distinct in vitro properties of embryonic and extraembryonic fibroblast-like cells are reflected in their in vivo behavior following grafting in the adult mouse brain. Cell Transplant.

[12] De Vocht N, Lin D, Praet J, Hoornaert C, Reekmans K, Le Blon D, Daans J, Pauwels P, Goossens H, Hens N (2013). Quantitative and phenotypic analysis of mesenchymal stromal cell graft survival and recognition by microglia and astrocytes in mouse brain. Immunobiology.

[13] Le Blon D, Hoornaert C, Daans J, Santermans E, Hens N, Goossens H, Berneman Z, Ponsaerts P (2014). Distinct spatial distribution of microglia and macrophages following mesenchymal stem cell implantation in mouse brain. Immunol Cell Biol.

[14] Praet J, Reekmans K, Lin D, De Vocht N, Bergwerf I, Tambuyzer B, Daans J, Hens N, Goossens H, Pauwels P (2012). Cell type-associated differences in migration, survival, and immunogenicity following grafting in CNS tissue. Cell Transplant.

[15] Praet J, Santermans E, Daans J, Le Blon D, Hoornaert C, Goossens H, Hens N, Van der Linden A, Berneman Z, Ponsaerts P. Early inflammatory responses following cell grafting in the CNS trigger activation of the sub-ventricular zone: a proposed model of sequential cellular events. Cell Transplant. 2014;24(8):1481–92. 10.3727/096368914X682800 25197881

[16] Reekmans K, De Vocht N, Praet J, Fransen E, Le Blon D, Hoornaert C, Daans J, Goossens H, Van der Linden A, Berneman Z, Ponsaerts P (2012). Spatiotemporal evolution of early innate immune responses triggered by neural stem cell grafting. Stem Cell Res Ther.

[17] Praet J, Guglielmetti C, Berneman Z, Van der Linden A, Ponsaerts P (2014). Cellular and molecular neuropathology of the cuprizone mouse model: clinical relevance for multiple sclerosis. Neurosci Biobehav Rev.

[18] Baekelandt V, Eggermont K, Michiels M, Nuttin B, Debyser Z (2003). Optimized lentiviral vector production and purification procedure prevents immune response after transduction of mouse brain. Gene Ther.

[19] Geraerts M, Michiels M, Baekelandt V, Debyser Z, Gijsbers R (2005). Upscaling of lentiviral vector production by tangential flow filtration. J Gene Med.

[20] Tambuyzer BR, Bergwerf I, De Vocht N, Reekmans K, Daans J, Jorens PG, Goossens H, Ysebaert DK, Chatterjee S, Van Marck E (2009). Allogeneic stromal cell implantation in brain tissue leads to robust microglial activation. Immunol Cell Biol.

[21] Bergwerf I, De Vocht N, Tambuyzer B, Verschueren J, Reekmans K, Daans J, Ibrahimi A, Van Tendeloo V, Chatterjee S, Goossens H (2009). Reporter gene-expressing bone marrow-derived stromal cells are immune-tolerated following implantation in the central nervous system of syngeneic immunocompetent mice. BMC Biotechnol.

[22] Praet J, Santermans E, Reekmans K, de Vocht N, Le Blon D, Hoornaert C, Daans J, Goossens H, Berneman Z, Hens N (2014). Histological characterization and quantification of cellular events following neural and fibroblast(-like) stem cell grafting in healthy and demyelinated CNS tissue. Methods Mol Biol.

[23] Reekmans K, De Vocht N, Praet J, Le Blon D, Hoornaert C, Daans J, Van der Linden A, Berneman Z, Ponsaerts P (2013). Quantitative evaluation of stem cell grafting in the central nervous system of mice by in vivo bioluminescence imaging and postmortem multicolor histological analysis. Methods Mol Biol.

[24] De Vocht N, Reekmans K, Bergwerf I, Praet J, Hoornaert C, Le Blon D, Daans J, Berneman Z, Van der Linden A, Ponsaerts P (2012). Multimodal imaging of stem cell implantation in the central nervous system of mice. J Vis Exp.

[25] Guglielmetti C, Praet J, Rangarajan JR, Vreys R, De Vocht N, Maes F, Verhoye M, Ponsaerts P, Van der Linden A (2014). Multimodal imaging of subventricular zone neural stem/progenitor cells in the cuprizone mouse model reveals increased neurogenic potential for the olfactory bulb pathway, but no contribution to remyelination of the corpus callosum. Neuroimage.

[26] Praet J, Orije J, Kara F, Guglielmetti C, Santermans E, Daans J, Hens N, Verhoye M, Berneman Z, Ponsaerts P, Van der Linden A (2015). Cuprizone-induced demyelination and demyelination-associated inflammation result in different proton magnetic resonance metabolite spectra. NMR Biomed.

[27] Orije J, Kara F, Guglielmetti C, Praet J, Van der Linden A, Ponsaerts P, Verhoye M (2015). Longitudinal monitoring of metabolic alterations in cuprizone mouse model of multiple sclerosis using 1H-magnetic resonance spectroscopy. Neuroimage.

[28] Benjamini Y, Hochberg Y (1995). Controlling the false discovery rate—a practical and powerful approach to multiple testing. J R Stat Soc Ser B Methodol.

[29] Liang KY, Zeger SL (1986). Longitudinal data-analysis using generalized linear-models. Biometrika.

[30] Thiessen JD, Zhang Y, Zhang H, Wang L, Buist R, Del Bigio MR, Kong J, Li XM, Martin M (2013). Quantitative MRI and ultrastructural examination of the cuprizone mouse model of demyelination. NMR Biomed.

[31] Le Blon D, Hoornaert C, Detrez J, Bevers S, Daans J, Goossens H, De Vos W, Berneman Z, Ponsaerts P. Immune remodelling of stromal cell grafts in the central nervous system: therapeutic inflammation or (harmless) side-effect? J Tissue Eng Regen Med. 2016. doi:10.1002/term.2188. 10.1002/term.2188 27320821

[32] Hoornaert CJ, Luyckx E, Reekmans K, Dhainaut M, Guglielmetti C, Le Blon D, Dooley D, Fransen E, Daans J, Verbeeck L (2016). In vivo interleukin-13-primed macrophages contribute to reduced alloantigen-specific T cell activation and prolong immunological survival of allogeneic mesenchymal stem cell implants. Stem Cells.

[33] Nessler J, Benardais K, Gudi V, Hoffmann A, Salinas Tejedor L, Janssen S, Prajeeth CK, Baumgartner W, Kavelaars A, Heijnen CJ (2013). Effects of murine and human bone marrow-derived mesenchymal stem cells on cuprizone induced demyelination. PLoS ONE.

[34] Salinas Tejedor L, Berner G, Jacobsen K, Gudi V, Jungwirth N, Hansmann F, Gingele S, Prajeeth CK, Baumgartner W, Hoffmann A (2015). Mesenchymal stem cells do not exert direct beneficial effects on CNS remyelination in the absence of the peripheral immune system. Brain Behav Immun..

[35] Yang MS, Park EJ, Sohn S, Kwon HJ, Shin WH, Pyo HK, Jin B, Choi KS, Jou I, Joe EH (2002). Interleukin-13 and -4 induce death of activated microglia. Glia.

[36] Miron VE, Boyd A, Zhao JW, Yuen TJ, Ruckh JM, Shadrach JL, van Wijngaarden P, Wagers AJ, Williams A, Franklin RJ, ffrench-Constant C (2013). M2 microglia and macrophages drive oligodendrocyte differentiation during CNS remyelination. Nat Neurosci.

[37] Skripuletz T, Hackstette D, Bauer K, Gudi V, Pul R, Voss E, Berger K, Kipp M, Baumgartner W, Stangel M (2013). Astrocytes regulate myelin clearance through recruitment of microglia during cuprizone-induced demyelination. Brain.

[38] Lampron A, Larochelle A, Laflamme N, Prefontaine P, Plante MM, Sanchez MG, Yong VW, Stys PK, Tremblay ME, Rivest S (2015). Inefficient clearance of myelin debris by microglia impairs remyelinating processes. J Exp Med.

[39] Guglielmetti C, Veraart J, Roelant E, Mai Z, Daans J, Van Audekerke J, Naeyaert M, Vanhoutte G, Delgado YPR, Praet J (2016). Diffusion kurtosis imaging probes cortical alterations and white matter pathology following cuprizone induced demyelination and spontaneous remyelination. Neuroimage.

